# Intestinal oxygen and microbiota crosstalk: implications for pathogenesis of gastrointestinal diseases and emerging therapeutic strategies

**DOI:** 10.1186/s13099-025-00783-4

**Published:** 2025-12-08

**Authors:** Tianze Shang, Rui Zhang, Yani Liu, Shaojun Shi

**Affiliations:** Department of Pharmacy, Union Hospital, Tongji Medical College, Huazhong University of Science and Technology, Wuhan, 430022 China

**Keywords:** Oxygen, Hypoxia, Gut microbiome, Mitochondria, Dysbiosis

## Abstract

The gut microbiota and its hypoxic host environment play a critical role in human health. Despite its importance, the mechanisms maintaining homeostasis and the characteristics defining dysbiosis remain largely undefined. In particular, the regulation of intestinal oxygen (IO) levels emerges as a critical factor in maintaining microbial balance. Host-driven factors, including epithelial oxygen consumption, mucosal perfusion, and luminal gas diffusion, establish a hypoxic gradient essential for the stable colonization by obligate anaerobes. Disruptions to this gradient, leading to pathological hyperoxia, are associated with overgrowth of facultative anaerobic bacteria and contribute to gastrointestinal diseases like ulcerative colitis, colorectal cancer, and irritable bowel syndrome. Emerging therapeutic approaches focus on modulating IO homeostasis to address dysbiosis. Compounds like sodium tungstate inhibit microbial respiratory pathways, while PPAR-γ agonists enhance mitochondrial efficiency in colonic epithelial cells, thereby restoring proper hypoxia. Dietary interventions and probiotic therapies also hold promise by promoting local anaerobic conditions and enhancing barrier functions, thus supporting the restoration of a healthy microbial community. This review highlights the role of IO in shaping host-microbe interactions, focusing on how host IO levels influence microbial homeostasis. We evaluate the potential for IO modulation to improve gut microbiota structure and explore its impact on microbial metabolism and disease pathogenesis. Additionally, we discuss the promise of dietary, probiotic, and pharmacological interventions in restoring the host’s control over the IO microenvironment and microbiota, aiming to prevent and treat related diseases.

**Supplementary Information:**

The online version contains supplementary material available at 10.1186/s13099-025-00783-4.

## Introduction

The advent of high-throughput sequencing in the 21 st century has significantly advanced human microbiome studies, particularly through the use of culture-independent methods to investigate host-associated microbial communities [1]. These studies have shown that microbiome imbalance, or dysbiosis, is associated with various diseases. As the largest microbial community in the body, the colonic microbiota produces metabolites that influence health. Thus, understanding the mechanisms that maintain gut homeostasis and the factors leading to its disruption during dysbiosis is crucial for addressing key questions in human microbiome research [2].

Over the past few decades, the focus of gut microbiota research has continued to evolve. Early studies on invertebrates identified abundant core microbial species, suggesting that defining a healthy gut microbiota might require identifying common core species. However, significant individual differences in human fecal microbiota made it impractical to define gut homeostasis and dysbiosis based on specific core species [3]. Moreover, even with large-scale data analysis from projects like the Human Microbiome Project [4], it remains unclear which features define gut homeostasis or its disruption [5]. These challenges indicate that high-throughput sequence analysis does not provide a straightforward path for defining healthy microbial communities.

Recently, it has been proposed that the host environment controls the healthy microbial community, offering a new approach to quantifying homeostasis [6]. According to this perspective, host-controlled environmental parameters are crucial for microbial growth on body surfaces. For example, oxygen levels in the intestinal lumen are a key driver of dysbiosis. Under physiological conditions, the intestinal lumen of mammals is hypoxic, which is associated with the intestinal epithelium, intestinal blood flow, and intestinal microbes [7]. This hypoxic gradient shapes the gut microbiota, promoting stable colonization by obligate anaerobes [8]. This shift in our understanding of dysbiosis provides a novel starting point for therapeutic strategies to restore microbiome health, suggesting that microbiome homeostasis can be assessed by measuring host functions, such as whether oxygen concentrations along the intestinal axis are within the normal range [9].

Herein, we summarize the role of intestinal oxygen (IO) in modulating host-microbe interactions, with emphasis on the mechanisms through which host IO levels shape microbial homeostasis. While other host factors, such as luminal pH and bile acids, also influence the gut ecosystem, IO remains a central regulator of microbial metabolism and spatial organization. We outline how altered IO reshapes microbial communities, drives metabolic defects, and contributes to intestinal disease. Distinct from previous reviews, we integrate recent advances in epithelial metabolism, HIF signaling, and oxygen regulation to provide a unified framework linking IO homeostasis (IOH), microbial ecology, and disease. Finally, we highlight emerging therapeutic strategies targeting different mechanisms to restore IOH, emphasizing their attractive translational potential.

## The host’s ability to restrict oxygen availability in the gut lumen

### Physiological basis of intestinal oxygen homeostasis (IOH)

Previous studies have found that the mammalian gut has a delicate oxygen gradient regulation system. In mammals, oxygen levels in the intestinal lumen decline longitudinally from the duodenum to the colon [10]. Oxygen-rich blood diffuses across capillary endothelial cells into intestinal epithelial cells, providing them with the oxygen they require. Consequently, the oxygen level in the duodenal lumen of mice is approximately 6% (45 mmHg O_2_), mirroring that found in blood vessels. Additionally, oxygen diffuses along a mucosa-to-lumen gradient, where it is subsequently consumed by luminal microbes. Thus, the partial pressure of oxygen in the colonic lumen is less than 10 mmHg. In general, factors such as blood perfusion in the intestinal mucosa, epithelial oxygen consumption, and luminal gas diffusion conspire to influence IO levels. Previous measurements of intestinal mucosa oxygenation are summarized in Table 1.

Intestinal mucosal perfusion and luminal oxygen diffusion are also important physiological bases for maintaining intestinal oxygen homeostasis. In the next section, the content of the lumen oxygen diffusion controlled by intestinal microbes will be detailed. Moreover, there is a countercurrent shunt phenomenon in the microcirculation of intestinal villi, which significantly affects the efficiency of oxygen transfer. Specifically, the distance between arterial and venous blood within the villi is approximately 20 μm [11]. This tight arrangement allows oxygen to diffuse directly from the arteries to the adjacent veins without reliance on red cell transport. Thus, a large amount of oxygen has been transferred to the venous system before blood reaches the tips of the villi, reducing the amount of blood oxygen received by the distal enterocytes [12].

**Table 1:** Measurements of intestinal mucosa oxygenation

Species	Experimental states	Method	Location	pO2	Reference
*Homo sapiens*	Patients undergoing laparotomy	a Clark-type-oxygen electrode	Ileum (serosal surface)	~ 34 mmHg	[13]
			Cecum (serosal surface)	~ 30 mmHg	
			Colon (serosal surface)	~ 39 mmHg	
	Healthy subjects		Rectum (luminal surface)	< 3 mmHg	[14]
*Mus musculus*	both Germfree and conventionally housed mice	OxyphorMicro probe	Cecum (luminal surface)	< 1 mmHg	[10]
			Cecum (tissue)	~ 40 mmHg	
	Physiological	electron paramagnetic resonance oximetry (EPR)	Colon (tissue)	~ 3–11 mmHg	[15]
			Duodenum (tissue)	~ 32 mmHg	
*Rattus norvegicus*	Baseline of a chronic mesenteric ischemia model	EPR	Small intestine (serosal surface)	~ 54.5 mmHg	[16]
*Sus scrofa domesticus*	Baseline of a hemorrhagic shock model	a Clark-type-oxygen electrode	Ileum (serosal surface)	~ 51 mmHg	[17]
			Colon (serosal surface)	~ 48 mmHg	
*Anas platyrhynchos domesticus*	Physiological	an oxygen electrode	Small intestine (luminal surface)	< 0.5 mmHg	[18]

### Epithelial oxygen consumption regulates IOH

Epithelial oxygen consumption is an important determinant of oxygen balance at the interface between host and environment. To support digestion and nutrient absorption, the intestinal epithelium requires a significant amount of oxygen to produce energy. Research has shown that the human colon’s oxygen consumption rate is approximately 8 µM·h^− 1^·cm^− 2^(*17*). An important source of energy for the intestinal epithelium includes mitochondrial oxidative phosphorylation (OXPHOS), which accounts for 79% of the ATP consumed by the Na-K-ATPase pump [19]. In brief, OXPHOS involves the oxidation of respiratory substrates in the mitochondrial matrix, producing NADH and FADH2. Protons and electrons derived from these molecules then pass through the mitochondrial respiratory chain, transferring to oxygen, which acts as the final electron acceptor, generating water ultimately [20].

Both in vitro and in vivo experiments have confirmed that inhibition of OXPHOS within mitochondria can significantly decrease oxygen consumption levels in intestinal tissue. For instance, Bohlen et al. [21] demonstrated that when mannitol solution was utilized to reduce sodium content in both small intestine tissues and corresponding mesenteric tissues of rats, there was an approximate 50% reduction in oxygen consumption within the intestinal mucosa due to inhibition of sodium ion transport activity. Consistent with these findings, Carra et al. [22]. Observed a 26% decrease in overall tissue oxygen consumption, after isolating mucosa from human colon tissue and treating it with a Na-K-ATPase inhibitor. In conclusion, host epithelial cells fuel the cells through a process of OXPHOS, which simultaneously consumes oxygen such that only trace amounts of oxygen cross the mucosal surface of the colon to the center of the lumen.

Moreover, the epithelial metabolic state establishes local oxygen gradients within the intestinal lumen, thereby shaping microbial spatial organization. Under physiological conditions, the low-oxygen lumen favors anaerobes, whose terminal metabolism produces butyrate—the preferred energy source for colonic epithelial cells. Kelly et al. [23] showed that antibiotic-treated mice exhibited elevated epithelial Po₂ and markedly reduced short-chain fatty acid (SCFA) levels, whereas luminal butyrate supplementation restored physiologic hypoxia and hypoxia-dependent signaling. In parallel, Pitt et al. [24] demonstrated that respiring *E. coli* membrane vesicles (MVs) ameliorated symptoms in a mouse model of gut inflammation, whereas MVs from cytochrome-deficient mutants failed to restore pimonidazole indices and SCFAs. Collectively, these findings indicate that epithelial OXPHOS activity is a key driver of luminal oxygen levels and microbial ecology.

### Hypoxia inducible factor (HIF)-mediated epithelial adaptation to hypoxia

Hypoxia inducible factor (HIF) is also closely related to physiological intestinal hypoxia. In the presence of oxygen, prolyl-4-hydroxylase domain enzymes (PHD1-PHD3) hydroxylate the α subunit of HIF (HIF1α, HIF2α and HIF3α) targeting it for ubiquitination via the VHL tumour suppressor protein (pVHL) and for subsequent proteasomal degradation [25]. When oxygen is insufficient to maintain PHD activity, HIF is stabilized, allowing it to translocate to the nucleus and regulate transcription of a broad group of genes important for cellular adaptation to hypoxia [26].

**Fig. 1:**
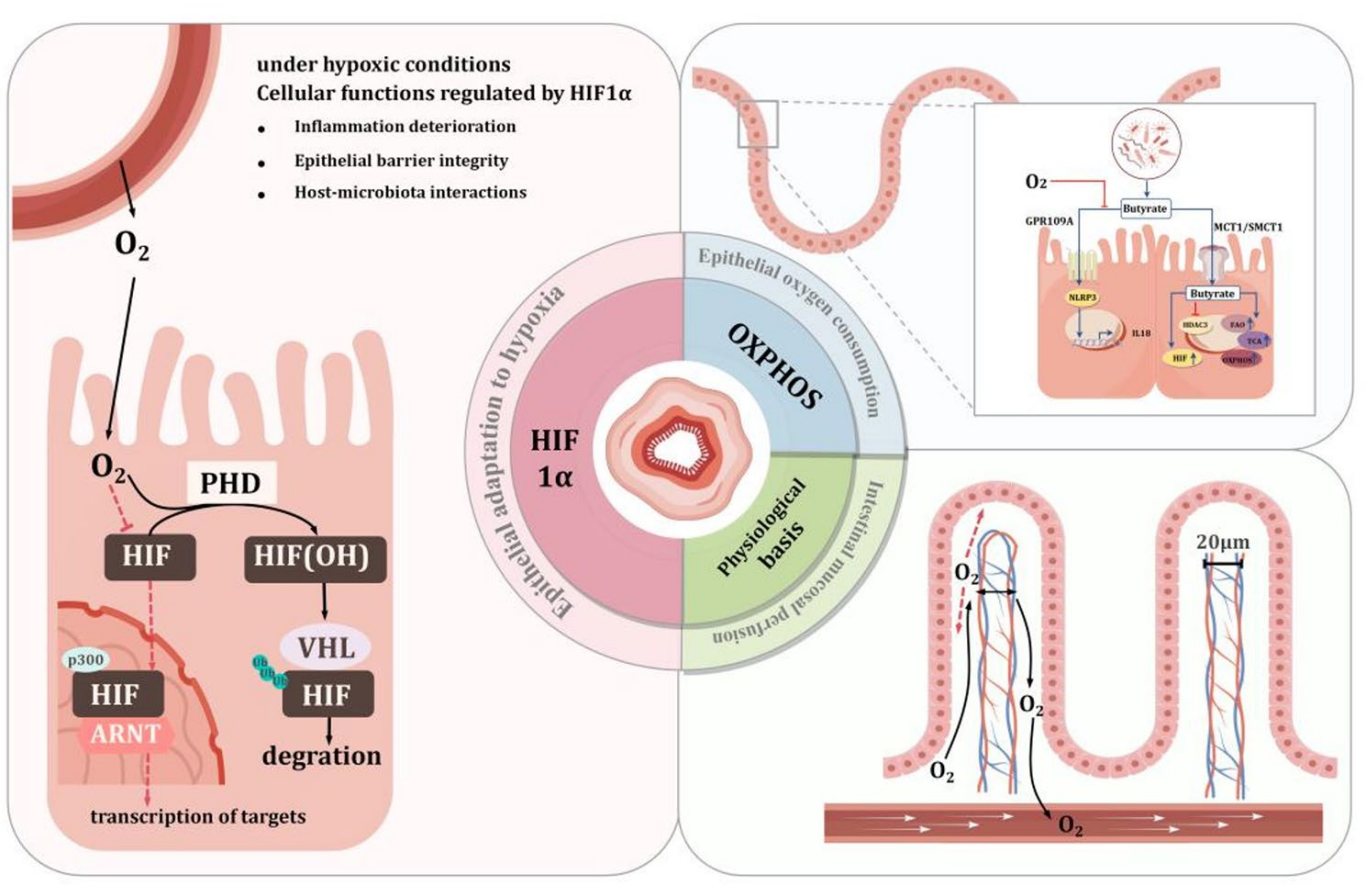
The host’s ability to restrict oxygen availability in the gut lumen

An expanding body of literature points to HIF as the key mediator of intestinal epithelial adaptation to its low oxygen microenvironment [27]. First, HIF promotes oxygen delivery by directly or indirectly regulating red blood cell production and distribution. Additionally, it has been reported that HIF regulates cellular metabolic adaptation to hypoxia, enabling the intestinal epithelium to utilize butyrate for oxidative metabolism by inhibiting pyruvate dehydrogenase and altering the tricarboxylic acid (TCA) cycle, thus providing an energy source [28, 29]. In summary, HIF is a key factor in intestinal epithelial adaptation to the hypoxic microenvironment and can promote oxygen delivery and hypoxic adaptation of intestinal epithelial cells. In short, results from in vitro and in vivo model systems have provided keen insight toward a better understanding of homeostatic physiology. Figure 1 illustrates the host’s ability to restrict oxygen availability in the gut lumen.

## Interactions between IOH and intestinal microbes

Of particular recent interest is the interplay between tissue oxygenation and the microbiota. Previous studies have established that the composition of the intestinal microbiome varies along the longitudinal axis of the gut and is related to the radial oxygen gradient distribution. Albenberg et al. [30] used phosphorescence quenching methods to directly demonstrate that host oxygenation affects intestinal lumen oxygenation. When the oxygenation of the host increases, intestinal lumen oxygenation also increases, indicating that oxygen diffuses from host tissues into the intestinal lumen to form a spatial oxygen gradient. Furthermore, after the host tissues recover their physiological state, the pO_2_ in the lumen decreases, and the abundance of aerobic microbes adhering to the mucosal surface increases. In summary, these phenomena not only demonstrate the close relationship between the host and the intestinal microbiome but also suggest that both are involved in regulating the local oxygen environment in the gut.

The intestinal bacteria use different energy metabolic pathways in aerobic and anaerobic conditions. In aerobic conditions, bacteria obtain energy through redox reactions. During this process, oxygen acts as an exogenous electron acceptor, substrates undergo phosphorylation or oxidative phosphorylation and ATP (adenosine triphosphate) is produced [31]. In anaerobic conditions, bacteria do not have exogenous electron acceptors and cannot complete the TCA cycle [32], mainly carrying out fermentation, releasing less energy than in aerobic conditions. During this process, pyruvate is converted into acetyl-CoA and formate by pyruvate methylhydroxylase, and then metabolized into CO_2_ and H_2_ by formate dehydrogenase. In summary, microorganisms that can maximize energy production will dominate the microbial community in which they live.

Furthermore, nitrate and oxygen serve as critical electron acceptors, enabling the host to precisely modulate the composition of the dominant bacterial community within the intestine by controlling the availability of these electron acceptors. For instance, in the hypoxic environment of the colonic lumen (~ 0.6% O_2_), obligate anaerobic primary fermenters predominantly utilize endogenous electron acceptors for their metabolic processes. These strict anaerobes, primarily represented by taxa within the classes *Bacteroidia (phylum Bacteroidetes)* and *Clostridia (phylum Firmicutes)*, form the backbone of the high-density microbial community in the colon [33]. Conversely, the ileum exhibits a slightly elevated average oxygen content compared to the colon (~ 1% O_2_), which correlates with an increased abundance of facultative anaerobic bacteria within its microbial community [34]. When oxygen becomes limiting, these bacteria switch to alternative electron acceptors such as nitrate to sustain respiratory metabolism [35].

Nitrate in the ileal lumen is mainly generated by NADPH oxidase 1 (NOX1) and inducible nitric oxide synthase (iNOS), which oxidize luminal or intracellular nitrogen compounds [28, 36]. During intestinal homeostasis, the average luminal nitrate concentration in the ileum of mice has been measured at approximately 6 mM [37]. In mice deficient in iNOS and NOX1 synthesis, a notable increase in the abundance of strictly anaerobic bacteria within the ileal microbiota has been observed, leading to a microbial profile that closely resembles that of the colonic microbiome [28]. Additionally, diet strongly modulates nitrate dynamics. High-fat or low-fiber diets can upregulate epithelial iNOS, elevate luminal nitrate, and favor Proteobacteria expansion, whereas fiber-rich diets enhance butyrate production, which suppresses nitrate synthesis via PPAR-γ activation [38].

**Fig. 2:**
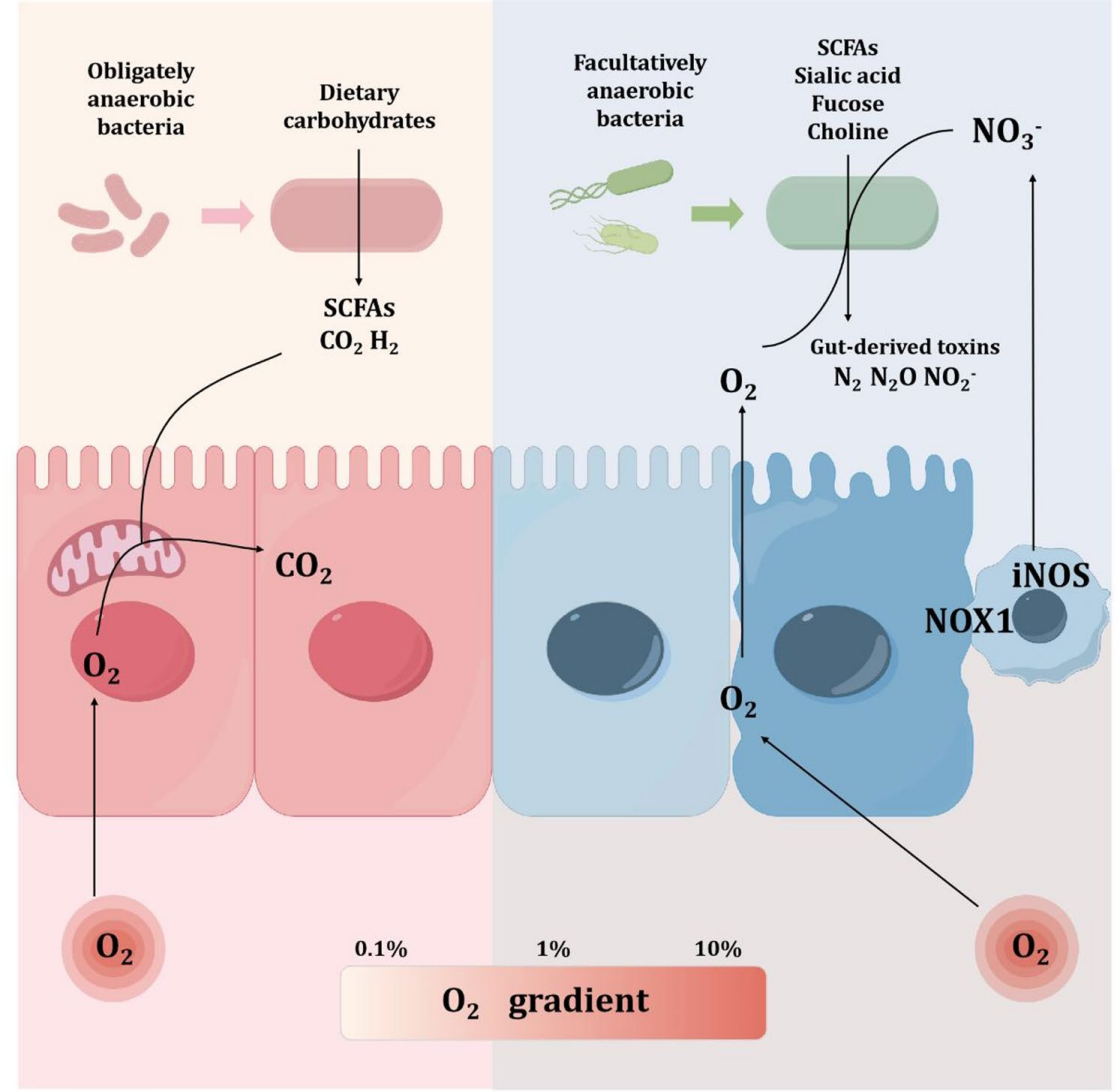
Intestinal oxygen gradients steer bacterial redox reactions

Beyond oxygen and nitrate, some inflammatory response products such as S-oxide, N-oxide, and formate also compete for bacterial respiration under microaerobic or inflamed conditions. For example, Enterobacteriaceae can use these byproducts to support their Dimethyl S-oxide respiration, trimethylamine N-oxide (TMAO) respiration and formate oxidation, providing a metabolic advantage when oxygen and nitrate are depleted [39]. While Bacteroidetes and Firmicutes bacteria lack the ability to utilize these by-products, which puts their growth at a disadvantage and ultimately leads to gut dysbiosis. Therefore, the balance among these alternative respiratory substrates plays a pivotal role in defining microbial community structure and metabolic output along the gastrointestinal tract. Figure 2 illustrates the interaction between gut microbiota and IOH.

## Oxygen battle in the gut links dysbiosis to diseases

As previously discussed, many human illnesses occur when the host is unable to properly regulate the IO microenvironment, including ulcerative colitis, colorectal cancer, graft-versus-host disease (GVHD) and irritable bowel syndrome (IBS). These diseases are all marked by dysbiosis, linked to an increased abundance of facultatively anaerobic bacteria in the colonic microbiota. Such organisms can use oxygen or nitrate as respiratory electron acceptors, thereby fueling their growth. Here, we aim to outline the major roles of pathological hyperoxia conditions of gut in the onset and progression of diseases. Impact of pathological hyperoxia on gut diseases is listed in Table 2.

**Table 2:** Impact of pathological hyperoxia on gut diseases

Model	Impact on diseases	Shifts of gut microbiome	Reference
TNBS-induced colitis mice	Compared to controls, colitis mice exhibited reduced mucosal hypoxia alongside increased neutrophil transmigration and impaired HIF stabilization.	Unknown	[40]
Mice infected with *Salmonella*	*Salmonella* overcomes colonization resistance by disrupting epithelial hypoxia. Pathogen outgrowth is fueled by catabolizing simple sugars, such as glucose, using a combination of aerobic respiration and mixed acid fermentation.	Obligate anaerobes ↓ - *Clostridia* ↓ - *Lachnospiraceae* ↓ - *Oscillospiraceae* ↓	[41]
DSS-induced colitis mice	DSS-induced colitis led to the loss of epithelial hypoxia, fostering an environment that promoted aerobic respiration and the dysbiotic expansion of commensal *E. coli*.	Facultative anaerobes ↑ - *Proteobacteria* ↑	[42]
Cachexia mice	In cachectic mice, expansion of *K. oxytoca* was linked to reduced PPAR-γ signaling, lower Pparg expression, and higher iNOS expression, accompanied by decreased β-oxidation (Cpt1a) and increased glycolysis (Hk2).	Obligate anaerobes ↓ - *Ruminococcaceae* ↓ - *Lachnospiraceae* ↓ - *Porphyromonadaceae* ↓ Facultative anaerobes ↑ - *Enterobacteriaceae* ↑ - *Klebsiella oxytoca* ↑	[43]
DSS-induced colitis mice	DSS treatment eliminated epithelial hypoxia and impaired mitochondrial bioenergetics (reduced ATP and PDH activity).	Facultative anaerobes ↑ - *Enterobacteriaceae* ↑	[44]
Mice on a high-fat diet	Epithelial hypoxia was eliminated in mice fed a high-fat diet, associated with reduced mitochondrial activity, lower ATP levels, and decreased PDH activity.	Facultative anaerobes ↑ - *Enterobacteriaceae* ↑	[45]
GVHD mice	After allo-HCT, disruption of epithelial OXPHOS reduced oxygen consumption, leading to higher oxygen levels, loss of physiological hypoxia, and microbiota dysbiosis.	Obligate anaerobes ↓ Facultative anaerobes ↑	[46]
Patients with IBS	Increased serum CRH concentration, mitochondrial damage in colonocytes, and gut dysbiosis.	Obligate anaerobes ↓ - *Methanobacteriaceae* ↓ - *Ruminococcus* ↓ Facultative anaerobes ↑ - *Pasteurellaceae* ↑ - *Haemophilus* ↑	[47]
Stress-induced IBS mice	IBS mice exhibited decreased OXPHOS activity, reduced ETS, and impaired complex IV activity. Stress-induced CRH release upregulated CRHR1 expression in colonocytes.	Obligate anaerobes ↓ - *Helicobacteraceae* ↓ - *Akkermansiaceae* ↓ Facultative anaerobes ↑ - *Moraxellaceae* ↑ - *Pseudomonadaceae* ↑	

### Ulcerative colitis

Ulcerative colitis (UC) is a multifactorial disease stemming from the impact of both environmental and genetic components on the intestinal microbiome [48]. From the host’s perspective, IOH plays a critical role in regulating inflammation associated with UC. For instance, epithelial cells from patients with ulcerative colitis exhibit decreased mitochondrial β-oxidation of butyrate to carbon dioxide [49], which is predicted to result in lower epithelial oxygen consumption. In this environment, the relative abundance of facultative anaerobic bacteria is expected to increase. For example, the fecal microbiota composition in patients with UC features an elevated abundance of facultatively anaerobic bacteria, including *Gammaproteobacteria* [50, 51]. Pathogenic *Enterobacterales* can exacerbate colitis in mouse models by competing for critical nutrients and leading to colonization resistance against enteric pathogens [52]. Furthermore, metabolic reprogramming of intestinal epithelial cells has been linked to UC. Virulent *Salmonella Typhimurium* can deplete short-chain fatty acids, resulting in a metabolic reprogramming of the intestinal epithelium that increases the availability of oxygen and nitrate in the mucus layer [41]. Moreover, Campbell et al. demonstrated that neutrophils modulate the host’s inflammatory response through localized depletion of O_2_ using a trinitrobenzene sulfonic acid (TNBS)-induced colitis model in mice [40]. In summary, UC is intricately linked to intestinal hypoxia condition, wherein oxygen levels serve as a primary determinant in the acceleration or inhibition of pathogen growth.

### Cancer

Gut microbiota dysbiosis, in particular the aerobic expansion of *Enterobacteriaceae* and altered gut barrier function, is one of the risk factors for the development of colorectal cancer (CRC) and cancer cachexia [53, 54]. One of the pathobionts implicated in causing CRC is colibactin-producing *Escherichia coli (E. coli)*, a facultatively anaerobic bacterium that exhibits an elevated fecal abundance in patients with CRC [55]. Consistent with this idea, Benign polyps developing early in life of patients with familial adenomatous polyposis are covered by patchy bacterial biofilms containing colibactin-producing *E. coli* [56], suggesting a link between early neoplasia of the colon and tumorigenic bacteria.

Restricting the bloom of Enterobacteriaceae decreased intestinal inflammation and reduced the incidence of colonic tumors in models of CRC [57]. Recently, studies have indicated that *Enterobacteriaceae* overrepresented in CRC may be due to their relative higher tolerance to oxygen diffused from the epithelium [58]. Cevallos et al. [42] suggested that an aerobic proliferation of *E. coli* is essential for the oncogenic activity of this pathobiont in a mouse model of CRC. Moreover, reduction in three butyrate-producing microbial families (*Ruminococcaceae*, *Lachnospiraceae* and *Porphyromonadaceae*) in cachectic mice promoting higher glycolysis and reducing β-oxidation, allows the emergence of *K.oxytoca*. and Enterobacteriaceae [43]. In conclusion, cancer-inducing activity of the microbiota is associated with increased epithelial oxygenation of the colon and an expansion of a prooncogenic driver species.

### Graft-versus-host disease (GVHD)

T cell-mediated gastrointestinal (GI) diseases such as graft-versus-host disease (GVHD) is a potentially fatal complication of allogeneic hematopoietic stem cell transplantation (allo-HSCT) [59]. In both human clinical biopsies and experimental allo-HSCT models, pathogenic T cells first target IECs, inducing mitochondrial dysfunction and suppression of OXPHOS activity [60]. Furthermore, the primary epithelial metabolic injury leads to diminished oxygen consumption and luminal oxygen leakage, thereby disrupting the physiologic hypoxic gradient that normally supports obligate anaerobes. As a consequence, reduction in intestinal microbial diversity with loss of obligate anaerobes such as *Akkermansia muciniphila* and *Escherichia coli*, is associated with increased GVHD mortality [61].

The alterations in host epithelial energy metabolism and gut microbiota suggest a potential link between microbiome dysbiosis and the development or progression of GVHD. Seike et al. [46] conducted studies utilizing SPF, antibiotic-treated, and germ-free animals to elucidate the roles of dysbiosis and IOH in GVHD. Their findings demonstrate that post-transplant dysbiosis was not the initiator but rather the outcome of epithelial oxygen dysregulation triggered by T cell-mediated tissue injury. Restoration of physiologic hypoxia through iron chelation preserved anaerobic commensals and attenuated GVHD pathology. However, it remains unclear whether targeting excess O_2_ or available drugs could serve as a novel clinical strategy to mitigate GVHD severity.

### Irritable bowel syndrome (IBS)

Irritable bowel syndrome (IBS) is a commonly encountered functional gastrointestinal disorder. Various factors may be involved in the pathogenesis of IBS, including impaired gut-brain interactions, gut microbiome dysbiosis and abnormal visceral sensation, etc [62]. By retrieving 16s-rRNA data of IBS patients and healthy controls [63, 64], researchers identified hub microbes that may play a vital role in the whole bacterial community of IBS individuals, including decreased levels of obligate anaerobes such as Methanobacteriaceae and *Ruminococcus*. In addition, the genus Streptococcus, as one of the members in aerobe groups, significantly increased in fecal samples and was also a potentially harmful microbe of IBS individuals. However, it is necessary to establish a comprehensive predictive model for IBS by integrating multiple variables, rather than using IBS-specific microbes alone.

More importantly, Zhang et al. [47] reported that fecal microbiota in patients with IBS display a compositional shift from obligate anaerobes, such as Methanobacteriaceae and *Ruminococcus*, toward facultative anaerobes, including Pasteurellaceae and *Haemophilus*, accompanied by a functional transition of microbial metabolism from anaerobic to aerobic processes. Consistent with human findings, mouse models of IBS exhibited reduced epithelial OXPHOS activity and diminished oxygen consumption in the colon, which subsequently triggered gut dysbiosis. Restoration of mitochondrial respiration ameliorated epithelial hypoxia and conferred metabolic resilience, thereby improving microbial imbalance. Collectively, these findings highlight a potential link between epithelial bioenergetics and microbial ecology in IBS.

## Possible ways to reduce abnormal intestinal oxygen concentrations

Building on prior discussions, the concept of host-microbiota interactions provides a framework for developing therapeutic strategies that modulate IOH to correct dysbiosis. In the following section, we review recent research on whether the adverse effects of dysbiosis can be mitigated by either inhibiting microbial respiratory pathways that drive community shifts or by enhancing host functions that restrict the availability of oxygen and nitrate in the gut lumen.

Among these strategies, pharmacological approaches have been most extensively investigated. Disruptions in colonic microbiota balance are frequently initiated by compromised mitochondrial function within the colonic epithelium, which perturbs local hypoxic conditions and luminal anaerobiosis [65]. Emerging evidence indicates that drugs that revive mitochondrial bioenergetics in the colonic epithelium can be used to restore gut homeostasis [66]. A key player in enhancing mitochondrial activity within the colonic epithelium is the nuclear receptor peroxisome proliferator-activated receptor gamma (PPAR-γ) [67]. Administration of PPAR-γ agonists, such as 5-aminosalicylic acid (5-ASA), has been shown to bolster mitochondrial efficiency specifically in colonic epithelial cells [45]. Both in patients and mice models of UC, treatment with 5-ASA normalizes the composition of the colonic microbiota by controlling the proliferation of facultative anaerobic bacteria [44, 68]. Additionally, in vitro studies have shown that activation of SIRTUIN 1 can enhance mitochondrial bioenergetics while concurrently lowering levels of circulating trimethylamine N-oxide (TMAO) [69–71]. These findings suggest that restoring epithelial hypoxia may also reduce the production of harmful microbial metabolites. However, these strategies were not initially designed to target IO regulation. Notably, the observed increase in facultative anaerobes and elevated levels of the luminal oxidative metabolite TMAO suggest that the beneficial effects of these interventions cannot be fully attributed to secondary anti-inflammatory actions. Rather, these findings imply that restoration of epithelial hypoxia may play a direct role in re-establishing IOH and in suppressing the generation of detrimental microbial metabolites.

Another pharmacological approach focuses on inhibiting microbial respiratory pathways. Sodium tungstate (Na_2_WO_4_) can limit the proliferation of facultative anaerobic bacteria by substituting for molybdenum in key respiratory enzymes [72], thereby preventing their overgrowth and improving IOH-related conditions. For instance, in mouse models of colorectal cancer, sodium tungstate treatment has been shown to inhibit the proliferation of colibactin-producing *E. coli*, leading to a reduction in polyp formation [57]. In UC models, tungstate selectively restricts Gammaproteobacteria expansion, reducing inflammation [73]. Additionally, limiting the availability of host-derived nitrate in the colonic lumen can prevent the production of harmful metabolites during gut dysbiosis. Aminoguanidine [71], a chemical inhibitor of iNOS, lowers serum TMAO levels in mice fed a high-fat diet with choline by blocking nitrate production. Targeting microbial metabolic pathways therefore represents a complementary strategy to restore intestinal ecological balance.

Finally, dietary interventions or oral probiotics have also been reported to hold great promise for IOH-associated GI disorders. Studies have shown that high-fat, low-fiber diets disrupt the host’s control over gut microbes [71, 74, 75], suggesting that dietary changes could help improve dysbiosis. Zhang et al. reported that supplementing broiler diets with yeast polysaccharides (YPS) increased jejunal villus height and enhanced antioxidant and barrier gene expression, thereby protecting against mycotoxin-induced injury [76]. Probiotic therapy also holds great promise for improving dysregulated gut microbiota. Yang et al. [77]. aggregated *Synechocystis* sp. PCC6803 (Sp) with *Bacillus subtilis* (BS) by biomimetic mineralization to form cyanobacteria-probiotics symbionts to efficiently regulate the gut microbiota and reshape the intestinal barrier functions in a murine model of acute colitis. Importantly, probiotics within the symbionts created a local anaerobic environment to activate the [NiFe]-hydrogenase enzyme of cyanobacteria, facilitating the production of H_2_ to persistently scavenge elevated reactive oxygen species and alleviate inflammatory factors. Although promising, these approaches remain largely preclinical and require further evaluation in human settings.

Different therapeutic strategies for modulating intestinal oxygen homeostasis vary widely in their levels of supporting evidence and translational readiness. Pharmacological interventions include approved agents with established safety profiles, such as 5-ASA. Although these drugs were not originally developed to target intestinal oxygen homeostasis, some clinical evidence supports their use in relevant contexts. Further clinical validation is needed for all strategies, with particular attention to safety, targeting, and long-term durability. In contrast, most dietary and probiotic interventions are supported mainly by preclinical evidence from animal models. To date, no clinical trials have directly evaluated probiotics or engineered microbial consortia for modulating intestinal oxygen homeostasis in humans. These approaches also face challenges such as uncertain colonization dynamics, host specificity, biosafety concerns, and regulatory barriers related to engineered strains. Nevertheless, regulating the microbiota through host-targeted approaches remains a promising direction because gut dysbiosis is closely linked to a broad spectrum of noncommunicable diseases. Table 3 summarizes potential strategies for reducing abnormal intestinal oxygen concentrations.

**Table 3:** Possible ways to reduce abnormal intestinal oxygen concentrations

Category	Intervention	Model	Mechanism of action	Reference
Pharmacological	5-ASA	Patients with UC	Activates epithelial PPAR-γ signaling to restore hypoxia; increases Firmicutes and decreases Proteobacteria, contributing to mucosal remission.	[44]
		DSS-induced colitis mice	Enhances epithelial oxygen consumption via PPAR-γ activation, restores epithelial hypoxia, and suppresses aerobic *E. coli* overgrowth.	[45]
Pharmacological	Resveratrol	Atherosclerosis mice	Remodels gut microbiota by increasing Lactobacillus and Bifidobacterium; inhibits TMA production, lowers TMAO levels, and enhances bile acid synthesis via FXR-FGF15 axis.	[70]
		High-fat diet mice	Restores epithelial hypoxia, downregulates epithelial Nos2 expression, and blunts diet-induced TMAO elevation.	[71]
Pharmacological	Sodium tungstate	Azoxymethane/DSS colitis mice	Replaces molybdenum in bacterial respiratory enzymes, selectively suppresses Enterobacteriaceae expansion, and reduces epithelial DNA damage.	[58]
		DSS colitis mice	Decreases Enterobacteriaceae load and mucosal inflammatory markers by inhibiting bacterial respiration.	[73]
Pharmacological	Aminoguanidine	High-fat diet mice	Inhibits host iNOS activity, reduces nitrate availability for facultative anaerobes, suppresses E. coli expansion, and lowers circulating TMAO levels.	[71]
Dietary	YPS	Broilers on mixed mycotoxin diets	Enhances intestinal barrier function and antioxidant capacity; reduces oxidative stress markers and hepatic toxin residues; improves villus structure and epithelial gene expression.	[76]
Probiotic	*Sp* + *BS* symbiont	Colitis mice	Establishes local anaerobic microenvironment; activates cyanobacterial [NiFe]-hydrogenase to generate H₂; scavenges ROS, modulates microbiota composition, and strengthens barrier integrity.	[77]

## Perspectives and future directions

Building on the preceding sections, growing evidence underscores the therapeutic potential of targeting IOH. However, several methodological and translational limitations continue to constrain progress. Establishing a physiological range of intestinal oxygen in healthy individuals would greatly aid the diagnosis of dysbiosis, yet such thresholds remain undefined. Current measurement techniques vary widely in spatial resolution and invasiveness, providing only partial or static information. Electrode- and probe-based methods detect local pO_2_ but fail to capture luminal heterogeneity [13, 14], whereas chemical markers visualize hypoxia ex vivo without temporal precision [78–80]. Moreover, gut factors such as enzymatic activity, bile acids, and dietary components can interfere with oxygen sensing, further complicating standardization [7]. The characteristics and potential clinical relevance of these approaches are summarized in Table 4.

Translational evidence in humans also remains limited. Most mechanistic insights into the relationship between epithelial bioenergetics, oxygen leakage, and microbial dysbiosis arise from animal models, leaving their human relevance uncertain. Quantitative human data correlating mucosal oxygen gradients with epithelial metabolism and microbial respiratory activity remain scarce. In addition, therapeutic strategies that modulate intestinal oxygen—such as PPAR-γ agonists, sodium tungstate, iNOS inhibitors, or engineered probiotics—require further validation regarding safety, tissue targeting, and durability of effect. Despite these challenges, IO regulation remains a promising direction for microbiome-based therapeutics. Metagenomic markers that track microbial respiratory gene abundance in feces may offer valuable non-invasive insights into IOH status and guide the development of oxygen-informed treatment strategies [52, 81].

**Table 4:** IO measurement technologies and their potential clinical relevance

Method	Advantages	Limitations	Potential Clinical Relevance	Reference
Clark-type electrode	Real-time; high sensitivity	Highly invasive; limited sampling area; low signal-to-noise ratio	Early intestinal Po_2_ profiling; mainly animal studies	[13]
Electron paramagnetic resonance (EPR) oximetry	Repeated; non-invasive; larger area measurement	Limited spatial resolution; probe ingestion needed; discrepancies with electrode	Luminal oxygen mapping; drug/dietary effect validation	[14]
Phosphorescence quenching probe	High accuracy; minimally affected by luminal contents	Requires specialized probes and optical equipment	Luminal oxygen mapping; drug/dietary effect validation	[78]
Pimonidazole/2-nitroimidazole	Stable hypoxia marker; visualizes gradients; viable tissue specific	Not real-time; requires staining; experimental	Detect hypoxic lesions; validate oxygen measurements; adjunct in therapy studies	[79]
Seahorse XF analyzer	Multiwell plate format; real-time measurement of cellular bioenergetics	In vitro only; specialized instrument	In vitro pharmacology; assessment of mitochondrial function and metabolic adaptation	[80]

## Conclusion

Intestinal oxygen serves as a central determinant of gut microbial composition and metabolic function. Disruption of IOH alters microbial respiration and redox balance, driving dysbiosis and promoting gastrointestinal pathologies. Although growing evidence underscores the therapeutic potential of targeting IOH, several barriers still hinder its clinical translation, including the lack of standardized, non-invasive methods to quantify IO and the limited causal understanding of host-microbe oxygen interactions in humans. Despite these challenges, preclinical studies consistently demonstrate that restoring IOH can re-establish microbial balance and improve mucosal resilience. Pharmacological, dietary, and probiotic strategies that modulate oxygen utilization or promote local anaerobiosis have shown encouraging results, underscoring the feasibility of oxygen-informed interventions. Future efforts should focus on establishing robust IO metrics, integrating redox readouts with multi-omics biomarkers, and validating targeted oxygen-based therapies in clinical settings to translate experimental insights into practical treatment strategies.

## Supplementary Information

Supplementary Material 1

## Data Availability

No datasets were generated or analyzed during the current study.
